# Using short read sequencing to characterise balanced reciprocal translocations in pigs

**DOI:** 10.1186/s12864-020-06989-x

**Published:** 2020-08-24

**Authors:** Aniek C. Bouwman, Martijn F. L. Derks, Marleen L. W. J. Broekhuijse, Barbara Harlizius, Roel F. Veerkamp

**Affiliations:** 1 Animal Breeding and Genomics, Wageningen University and Research, P.O. Box 338, 6700 AH Wageningen, The Netherlands; 2 Topigs Norsvin Research Center, 6640 AA Beuningen, The Netherlands

**Keywords:** Karyotype, Pig, Reciprocal translocation, Whole genome sequencing

## Abstract

**Background:**

A balanced constitutional reciprocal translocation (RT) is a mutual exchange of terminal segments of two non-homologous chromosomes without any loss or gain of DNA in germline cells. Carriers of balanced RTs are viable individuals with no apparent phenotypical consequences. These animals produce, however, unbalanced gametes and show therefore reduced fertility and offspring with congenital abnormalities. This cytogenetic abnormality is usually detected using chromosome staining techniques. The aim of this study was to test the possibilities of using paired end short read sequencing for detection of balanced RTs in boars and investigate their breakpoints and junctions.

**Results:**

Balanced RTs were recovered in a blinded analysis, using structural variant calling software DELLY, in 6 of the 7 carriers with 30 fold short read paired end sequencing. In 15 non-carriers we did not detect any RTs. Reducing the coverage to 20 fold, 15 fold and 10 fold showed that at least 20 fold coverage is required to obtain good results. One RT was not detected using the blind screening, however, a highly likely RT was discovered after unblinding. This RT was located in a repetitive region, showing the limitations of short read sequence data. The detailed analysis of the breakpoints and junctions suggested three junctions showing microhomology, three junctions with blunt-end ligation, and three micro-insertions at the breakpoint junctions. The RTs detected also showed to disrupt genes.

**Conclusions:**

We conclude that paired end short read sequence data can be used to detect and characterize balanced reciprocal translocations, if sequencing depth is at least 20 fold coverage. However, translocations in repetitive areas may require large fragments or even long read sequence data.

## Background

In diploid mammals, a normal karyotype consist of two copies of each chromosome. Unfortunately, various unbalanced constitutional abnormal karyotypes have been observed in unviable offspring and individuals with various clinical disorders. In contrast, balanced constitutional chromosomal abnormalities usually result in viable individuals with no apparent phenotypical consequences, except for reduced fertility. Balanced chromosomal abnormalities, such as reciprocal translocations (RT), have no gain or loss of chromosomal material. Hence, all required genes to function properly are present, unless the breakpoints disrupt such genes.

The prevalence of chromosomal abnormalities in pigs is estimated to be 0.47% [1], but varies in different countries [2–4]. Most reported chromosomal abnormalities are RT, where parts of non-homologous chromosomes are exchanged. They can be inherited from a parent or occur de novo in the meiosis. Heterozygous individuals carrying a balanced RT produce three type of gametes: unbalanced gametes, balanced RT carrying gametes, and normal gametes. The unbalanced gametes give rise to embryos carrying partial monosomy and trisomy, which are mostly lethal or result in severely malformed piglets [1, 5, 6]. Carriers of balanced RTs tend to be hypoprolific individuals with a reduction in litter size of 10–100%, and on average approximately 40% [1, 3, 4, 7]. Via artificial insemination (AI) such RTs can quickly spread in a population, resulting in severe economic losses [3], therefore modern pig breeding companies perform karyotype screening for the boars intended for usage in AI.

Currently, chromosome staining of these young boars is performed for karyotype screening. This requires fresh blood samples to culture, which is logistically complex and a labour intensive process subjected to considerable variability. An important limitation of current routine chromosome staining is the fact that not all chromosomal rearrangement can be detected. For instance, translocations smaller than the band size (~ 3-10 Mb) or exchange of parts similar in size and banding pattern remain undetected [5]. In addition, chromosome staining does not indicate the exact breakpoint position, and does not allow further investigation of the breakpoint area.

Standard SNP-array genotyping can be used to routinely screen for unbalanced karyotypes using the intensity information, because there is gain or loss of DNA [5, 8, 9]. However, SNP-arrays cannot be used to detect balanced RTs, because the SNP is present in its expected two copies, but the position in the genome has changed. With SNP genotyping it is unknown in which order they appeared on the chromosome unless the linkage phase is known, e.g. in karyomapping [10]. Karyomapping is used as a preimplantation genetic test for embryos of balanced RT carriers [10]. This is very suitable for screening offspring from known RT carriers, but not applicable for routine screening of balanced karyotype abnormalities in boars before entering AI and produce offspring. In contrast, whole genome sequencing techniques result in reads that provide the base pair sequence of fragments of the chromosomes. Human studies, especially cancer research, have shown that balanced RTs can be detected using a combination of split alignments (parts of the same read map to two different locations on the reference genome) and discordantly mapped read pairs (paired ends that were not aligned to the reference genome within the expected distance or orientation). Bioinformatic tools that use a combination of split reads and discordant pairs are most successful and precise in detection of balanced RTs [11]. Currently, there are three types of sequence data that have been used for balanced RT detection in humans: long read, linked-read, and short read sequence data. Long read sequencing is a rather expensive technique at the moment, but has been most successful at detection of complex rearrangements and breakpoints located in repetitive elements, as well as for simple RT. [12, 13] Short reads sequencing is relatively cheap and performs well at detecting simple rearrangements (e.g. [14–16]). While linked-reads (e.g. 10x Genomics) seem promising, especially for cases in repetitive elements, the technique and detection tools need further development [17].

In general, sequence reads will not only enable detection of RTs, but also enhance refinement of the location of the breakpoints and detailed description of the breaks and junctions. In pigs, Grahofer et al. [5] have successfully refined the breakpoint location of an segregating RT causing malformed piglets using sequence data of an unbalanced and balanced sib. Donaldson et al. [18] showed that translocation breakpoints are nonrandomly distributed across cytogenetic bands, they are more prevalent on longer cytogenetic bands, gram negative bands, and common fragile sites. Investigating the sequence around breakpoints will enhance the detection and characteristics of translocation hotspots in the genome.

Envisioning a further reduction of sequencing cost and more extensive use of sequence data in livestock, it might become routine to sequence the selection candidates of breeding programs. Being able to use these sequences for screening of karyotype abnormalities is cost effective and might even be a better screening than the currently applied chromosome staining. Therefore the aim of this study was to test the possibilities of paired end short read sequencing for detection of balanced RTs at different sequencing coverage depth and investigate the breakpoints and junctions of the translocations using the sequence information.

## Results

**Fig. 1:**
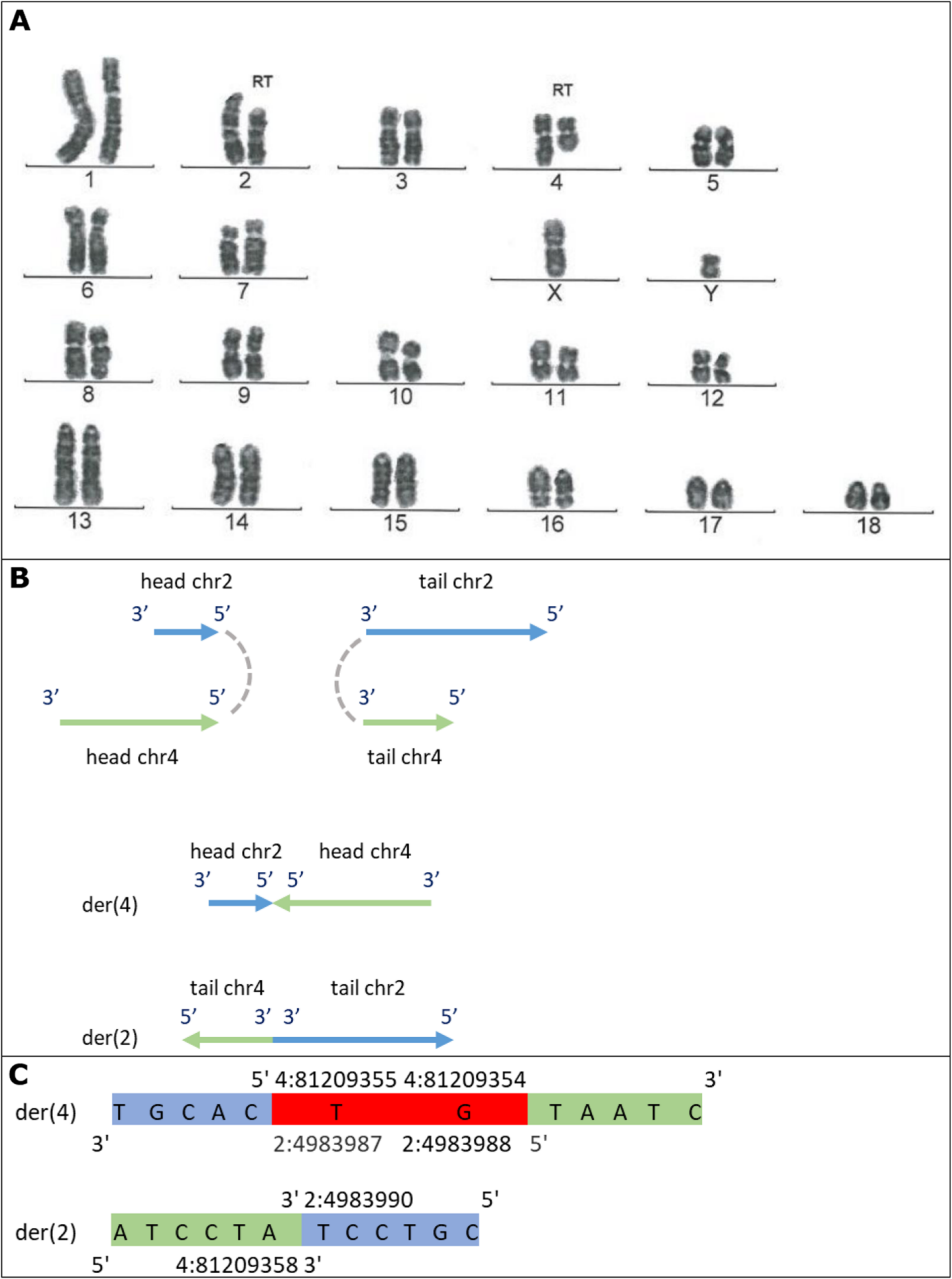
Representation of t (2, 4). **a** Giemsa stained karyotype picture of t (2, 4) carrier (Pig 3 in this case, but is the same for Pig 1, 4 and 6). The derived chromosomes are indicated with the note ‘RT’. **b** Graphical representation of the double stranded break and connection type, and the derived chromosomes (der). Head and tail are the two resulting ends of the chromosome due to the breakpoint, with head being the top part (start of chromosome till breakpoint), and tail the bottom part (breakpoint till end of chromosome). The grey dashed lines show how the junctions were created, here 3′ to 3′ and 5′ to 5′. **c** The sequence of the derived chromosomes at the junction. Red sequence indicates the overlap (microhomology) in sequence at the junction. In both **b** and **c** chromosome 2 is in blue and 4 in green

**Fig. 2:**
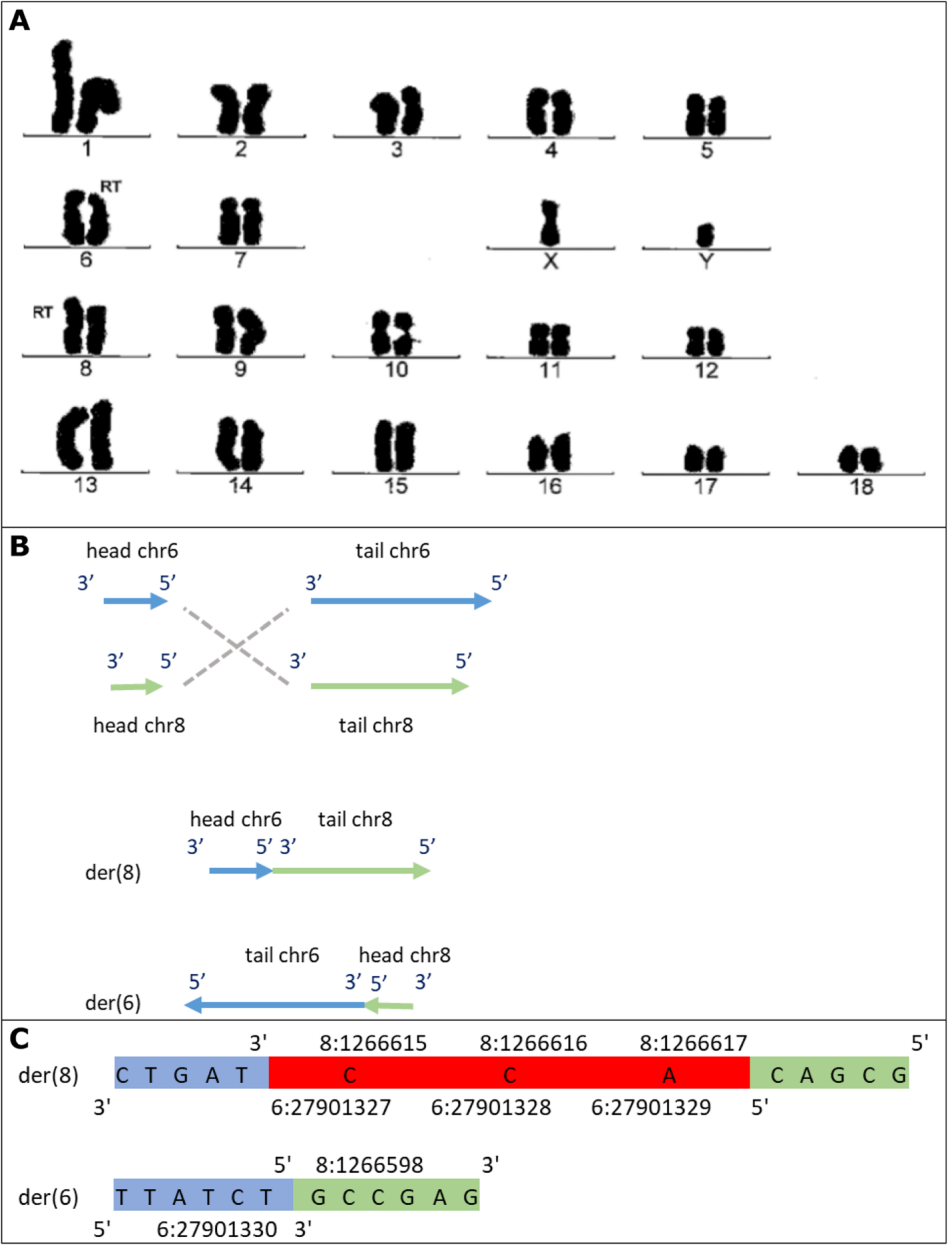
Representation of t (6, 8). **a** Giemsa stained karyotype picture of t (6, 8) carrier (Pig2). The derived chromosomes are indicated with the note ‘RT’. **b** Graphical representation of the double stranded break and connection type, and the derived chromosomes (der). Head and tail are the two resulting ends of the chromosome due to the breakpoint, with head being the top part (start of chromosome till breakpoint), and tail the bottom part (breakpoint till end of chromosome). The grey dashed lines show how the junctions were created, here 3′ to 5′ and 5′ to 3′. **c** The sequence of the derived chromosomes at the junction. Red sequence indicates the overlap (microhomology) in sequence at the junction. In both **b** and **c** chromosome 6 is in blue and 8 in green

**Fig. 3:**
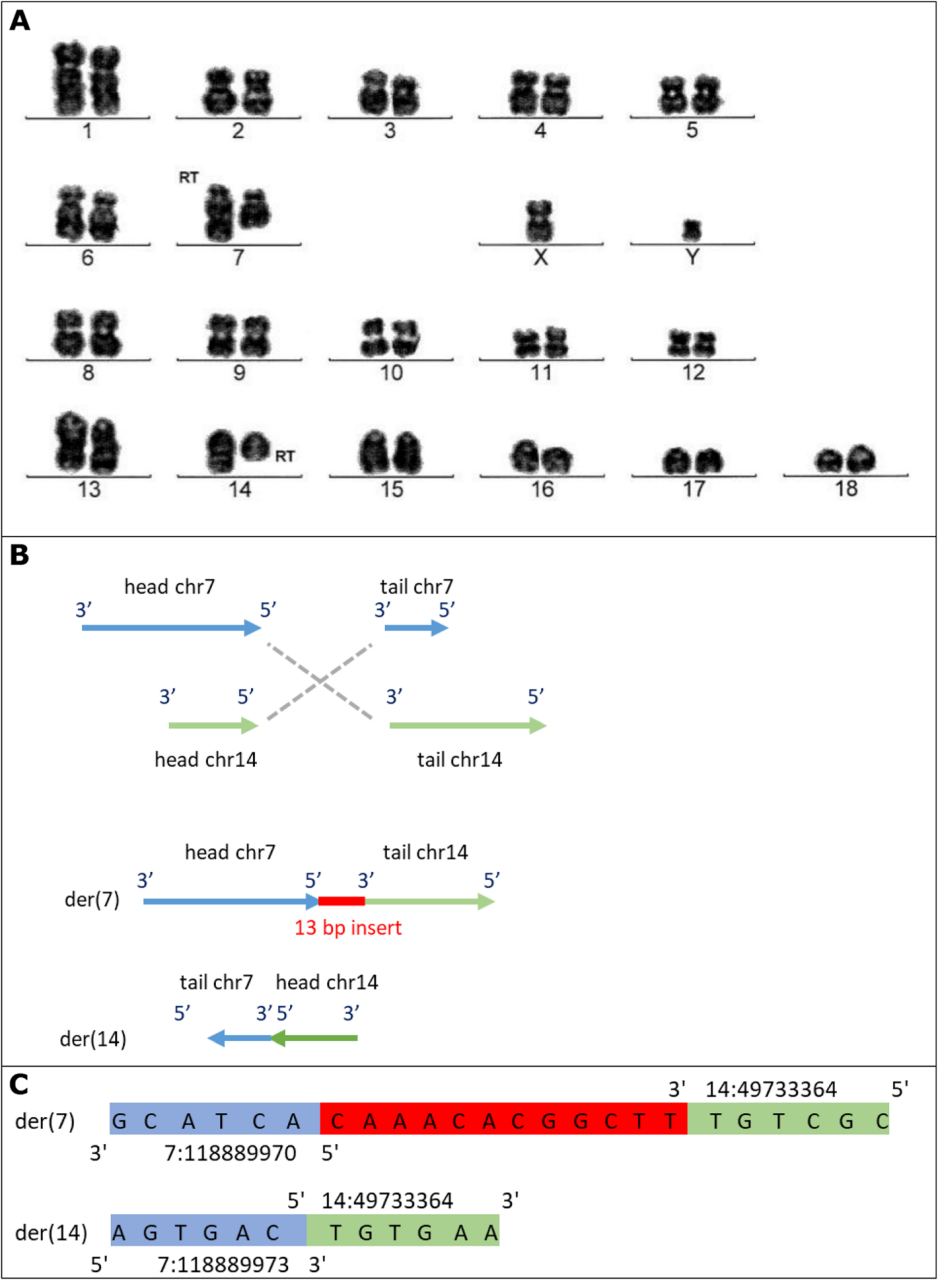
Representation of t (7, 14). **a** Giemsa stained karyotype picture of t (7, 14) carrier (Pig5). The derived chromosomes are indicated with the note ‘RT’. **b** Graphical representation of the double stranded break and connection type, and the derived chromosomes (der). Head and tail are the two resulting ends of the chromosome due to the breakpoint, with head being the top part (start of chromosome till breakpoint), and tail the bottom part (breakpoint till end of chromosome). The grey dashed lines show how the junctions were created, here 3′ to 5′ and 5′ to 3′. **c** The sequence of the derived chromosomes at the junction. Red sequence indicates the inserted sequence (micro-insertion) at the junction. In both **b** and **c** chromosome 7 is in blue and 14 in green

**Fig. 4:**
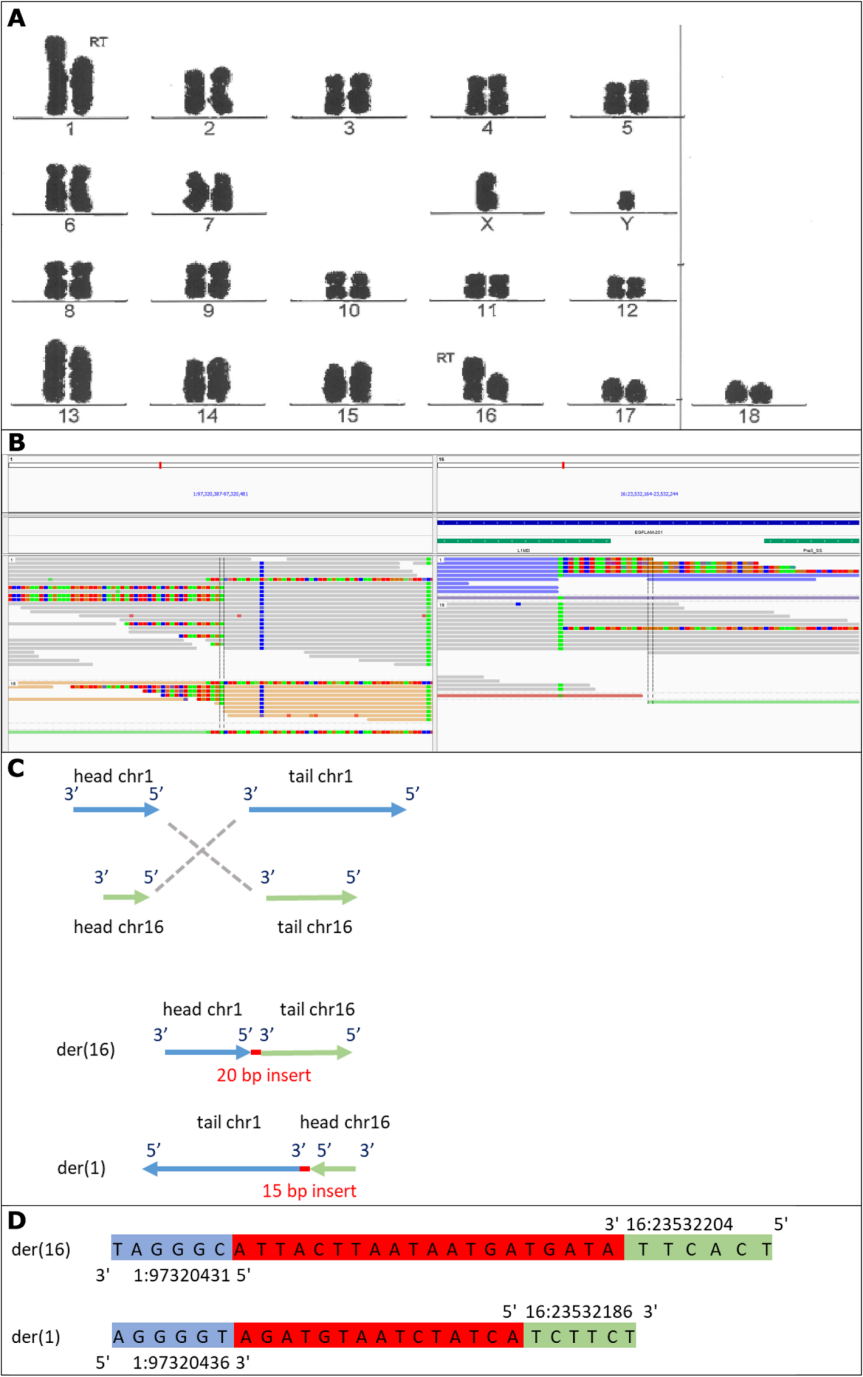
Representation of t (1, 16). **a** Giemsa stained karyotype picture of t (1, 16) carrier (Pig7). The derived chromosomes are indicated with the note ‘RT’. **b** Breakpoint locations on chromosome 1 and 16 visualized in IGV with gene (blue) and repeat track (green) at top. Grey read are normal reads. Colored reads are reads of a discordant pairs (mate maps to another chromosome). Green (A), red (T), blue (C) and orange/brown (G) show mismatched bases from split reads. **c** Graphical representation of the double stranded break and connection type, and the derived chromosomes (der). Head and tail are the two resulting ends of the chromosome due to the breakpoint, with head being the top part (start of chromosome till breakpoint), and tail the bottom part (breakpoint till end of chromosome). The grey dashed lines show how the junctions were created, here 3′ to 5′ and 5′ to 3′. **d** The sequence of the derived chromosomes at the junction. Red sequence indicates the inserted sequence (micro-insertion) at the junction. In both **c** and **d** chromosome 1 is in blue and 16 in green

For this study, DNA material from 7 male RT carriers was available. These RT carriers were detected during routine karyotype screening of AI boars with chromosome staining. The Giemsa stained pictures of the karyotype of the carriers are in Figs. 1a, 2a, 3a and 4a, with the RT position indicated.

### Pedigree and litter size

The pedigree and average litter size (number of liveborn piglets) of parents were evaluated to determine if the RT was de novo or inherited, and if carriers were showing the expected reduction in litter size. Pig 1, 3, 4, and 6 were related, they all carried the same RT: t (2;4). The other pigs were unrelated and carried the following RTs: t (6;8), t (7;14), t (1;16).

Except for pig 7, all the RT carriers studied here had relatives reported to carry the same RT, suggesting they were not de novo but inherited from a parent. The expectation is that RT carriers have reduced number of live born litter sizes because half of the gametes they produce are unbalanced, leading to unbalanced foetuses, which are most likely unviable. Therefore, we had a look at the litter size of the RT carriers and their sires and dams (Table 1). The RT carriers belong to a boar line with an average litter size of 10 piglets. In general, the sires showed a higher average litter size than the dams, because for the sires also crossbred litters were included. In addition, the sire averages were based on much larger number of litters and hence showed lower standard deviations. Table 1 shows that pig 7 sired litters himself (29 in total, but only 6 with litter size records) and showed a reduced average live born litter size of 5 (±2.6). Given an average litter size of 10 for this boar line, this suggests that indeed half of the litter was unviable. Also the sires of Pig 5 and 7 had a reduced litter size, suggesting these sires are carriers of the respective RT and the RT was inherited rather than de novo. The dam of pig 2 had and average litter size of 8 with a large standard deviation. She gave birth to only 2 litters, one with 13 liveborn and 2 stillborn piglets, and the second with only 3 liveborn piglets of which pig 2 was one. The sire of pig 2 showed average litter size (14.0 ± 3.9) based on a large number of litters (275), suggesting the RT was inherited from the dam. Which can be supported by the fact that she gave birth to another known RT carrier in her first litter (i.e. maternal half sib of pig 2).

**Table 1:** Litter size and number of litters with records for the studied individuals and their parents

	Individual		Sire		Dam	
	Litter size^a^	N^b^	Litter size	N	Litter size	N
Pig1	–	0	12.2 (±3.7)	174	10.4 (±3.3)	5
Pig2	–	0	14.0 (±3.9)	275	8 (±7.1)	2
Pig3	–	0	14.4 (±3.9)	211	10.5 (±2.4)	6
Pig4	–	0	14.4 (±3.9)	211	10.5 (±2.4)	6
Pig5	–	0	6.6 (±2.9)	13	9.4 (±1.6)	7
Pig6	–	0	12.0 (4.0)	55	10.5 (±2.4)	6
Pig7	5 (±2.6)	6	6.5 (±2.4)	6	8.5 (±1.7)	4

^a^ Litter size based on liveborn piglets only

^b^ N is the number of litters with recorded litter size

Pig 1 was a maternal half sib of the dam of pig 3, 4, and 6. Pig 3 and 4 were littermates, and pig 6 was a maternal half sib of them, see pedigree graph in Supplementary Figure 1. They all carried the same RT, t(2;4), which they most likely inherited from their (untested) (grand)mother. Surprisingly, for pig 1, 3, 4, and 6 there was no clear reduction in litter size from their parents. Given their pedigree relation, the dam of pig 3, 4, and 6, and dam of pig1 (maternal granddam of pig 3, 4, and 6) are most likely carriers of t (2, 4) (Supplementary Figure 1). However, these dams had an average live born litter size of 10.5 (±2.4) and 10.4 (±3.3), respectively, and none of the sires involved showed a reduced litter size.

These litter sizes and pedigree relations suggest that all studied RT carriers inherited the RT from a parent rather than a de novo occurrence of the RT.

### Blind detection of RT in short read sequence data

**Fig. 5:**
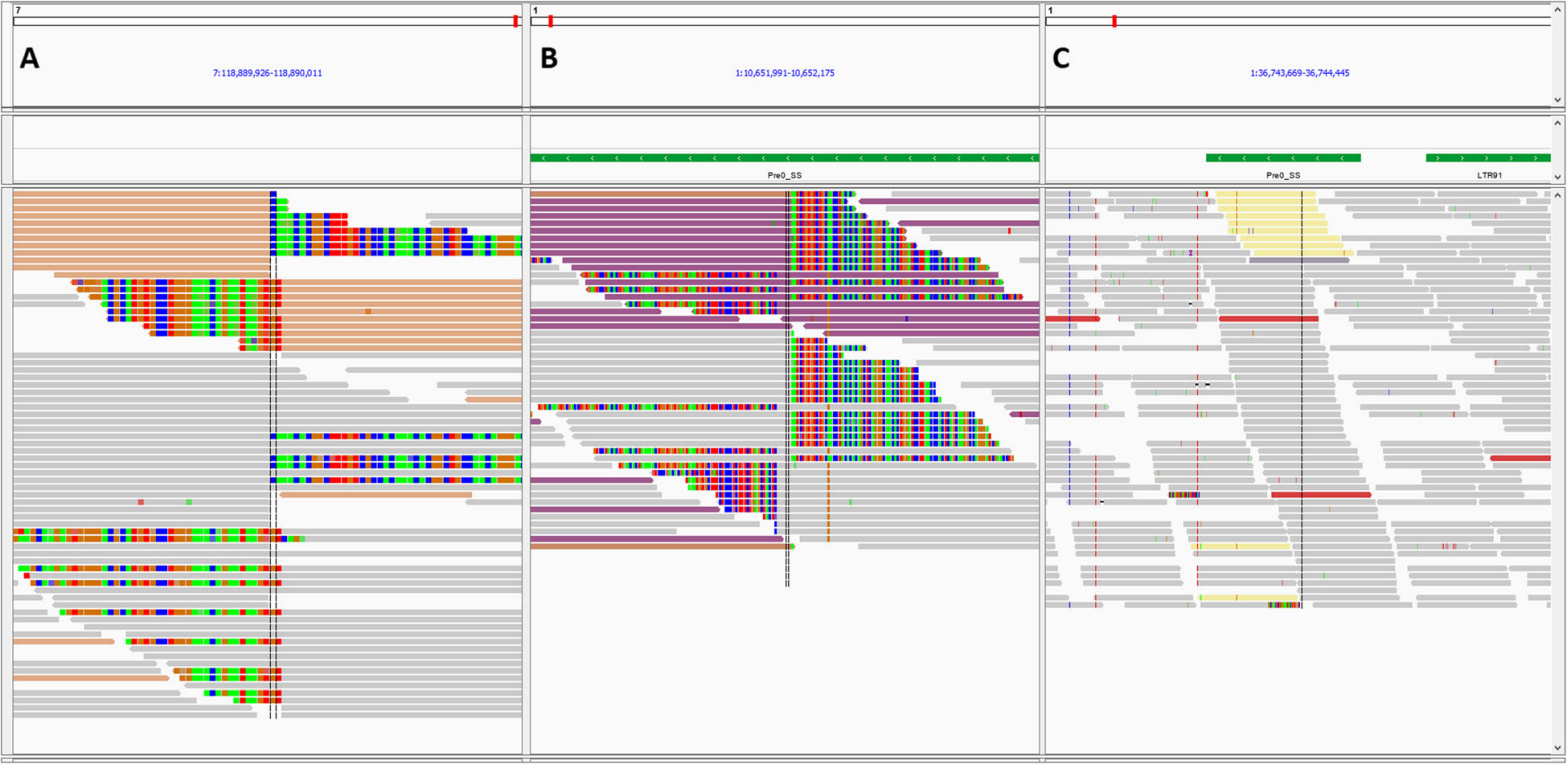
Three types of breakpoints observed during visual inspection of the aligned reads using IGV. Grey reads are normal reads. Colored reads are reads of discordant pairs (mate maps to another chromosome). Green (A), red (T), blue (C) and orange/brown (G) bases show mismatched bases from split reads. **a** Possible RT breakends showing normal reads of the intact chromosome as well as discordant read pairs and split reads from the affected chromosomes. **b** Non RT event due to a repetitive element, showing only discordant and split reads, good reads from the intact chromosome are lacking. **c** Non RT event due to repetitive element, showing forward and reversed discordant read pairs in same area

The DNA material of the RT carriers was sequenced at 30 fold coverage and screened for inter-chromosomal breakpoints using the structural variant caller DELLY. After filtering, the remaining translocations required further classification into none RT events and possible RT translocation by visualising the breakpoints in IGV [19]. Figure 5 shows three types of breakpoints observed, with possible RT translocations showing good reads of the intact chromosome as well as discordant read pairs and split reads at the two breakends from the affected chromosome (Fig. 5a). A true RT should show two patterns like Fig. 5a, one on each chromosome involved. The other two observed patterns, Fig. 5b and c, were typical for repetitive elements.

Table 2 shows the resulting number of inter-chromosomal translocations from each step in our analysis. After visual inspection, we correctly discovered 6 out of the 7 RTs in a blind analysis (IGV images of the aligned reads on chromosome regions involved are given in Supplementary Figure 2). The t (1;16) from pig7 was not detected in the blind analysis. All final detected RTs involved the chromosomes that match the RT results from karyotype staining. Therefore, we are confident these are the actual breakpoints causing the RT. For the related pigs with t (2;4), we detected the exact same translocation positions in all 4 pigs. The 15 non-carrier animals all came out negative using the same filtering criteria and visual inspection as the carriers (Table 2).

**Table 2:** Translocation output at various stages of the pipeline for 7 carriers and 15 non-carriers with negative RT results

Animal	Karyotype	Coverage	DELLY out	Basic filt	Final filt^a^	Visual insp
Pig1	t(2;4)	32.6	59,865	1127	30 (15)	1 (2,4)
Pig2	t(6;8)	37.9	80,967	1426	56 (28)	1 (6,8)
Pig3	t(2;4)	33.4	70,794	1185	34 (17)	1 (2,4)
Pig4	t(2;4)	35.1	78,454	1175	38 (19)	1 (2,4)
Pig5	t(7;14)	37.2	79,418	1503	68 (34)	1 (7,14)
Pig6	t(2;4)	31.1	61,639	1067	38 (19)	1 (2,4)
Pig7	t(1;16)	30.3	64,583	1116	44 (22)	0(FN^b^)
Contr1	normal	33.5	70,326	1281	58 (29)	0
Contr2	normal	31.7	58,678	974	36 (18)	0
Contr3	normal	30.3	68,711	1009	32 (16)	0
Contr4	normal	28.8	42,268	788	24 (12)	0
Contr5	normal	30.7	70,738	1116	30 (15)	0
Contr6	normal	34.3	69,866	1295	50 (25)	0
Contr7	normal	31.2	49,269	873	32 (16)	0
Contr8	normal	29.1	66,194	1145	38 (19)	0
Contr9	normal	29.2	62,080	1118	34 (17)	0
Contr10	normal	28.9	41,329	556	16 (8)	0
Contr11	normal	29.4	48,292	810	34 (17)	0
Contr12	normal	29.1	65,296	1085	34 (17)	0
Contr13	normal	29.3	52,289	868	36 (18)	0
Contr14	normal	29.0	55,121	920	46 (23)	0
Contr15	normal	29.2	58,460	942	36 (18)	0

^a^ one of the filter criteria for the detected inter-chromosomal translocations was the presence of a matching pair, hence between brackets are the number of reciprocal pairs, i.e. the actual number of possible RTs

^b^ *FN* False negative

### Analysis of breakpoints and junctions

The sequence data made it possible to refine the RT location and to investigate the breakpoints and junctions of the three detected RTs. For the t (2;4) translocation, we had four related individual showing the exact same breakpoints and junctions at 2:4983988/ 4:81209353 and 2:4983990/ 4:81209358, confirming the RT was inherited. Both breakpoints were located within a gene. The break on chromosome 2 was located in an intron of ENSSSCG00000032003, which is an ortholog of the human BRCA2 gene, a breast cancer gene involved in DNA repair (OMIM entry 600,185 [20];). The break on chromosome 4 was located in an exon of C1orf112, which is an uncharacterized open reading frame (Supplementary Figure 2).

For t (2;4), the breakends connected 5′ to 5′ (head of chr2 to head of chr4) and 3′ to 3′ (tail of chr2 to tail of chr4), see Fig. 1b for schematic representation. At the 3′ to 3′ junction there was a blunt end ligation. The 5′ to 5′ junction showed microhomology, i.e. there was an overlap of 2 bp in the sequence of both breakends, at 2:4983987–4,983,988 and 4:81209355–81,209,354 (Fig. 1c). From chromosome 2 one base was lost (2:4983989). From chromosome 4, two bases were lost (4:81209356–81,209,357). A normal copy of chromosome 2 has a length of 151.9 Mb, while a normal copy of chromosome 4 is 130.9 Mb long. The 3′ to 3′ junction resulted in a chromosome length of 196.7 Mb (147.0 Mb from chromosome 2 and 49.7 Mb from chromosome 4), matching the larger derived chromosome 2 in the karyotype picture (Fig. 1a). The 5′ to 5′ junction resulted in a chromosome length of 86.2 Mb (5.0 Mb from chromosome 2 and 81.2 Mb from chromosome 4), matching the smaller derived chromosome 4 in the karyotype picture (Fig. 1a).

We mapped the region of the t (6;8) translocation to 6:27901326/ 8:1266615 and 6:27901330/ 8:1266598. Both breakpoints were located within a gene. The breakpoint on chromosome 6 was located in an intron of SLC9A5, which is involved in pH regulation to eliminate acids generated by active metabolism or to counter adverse environmental conditions. The breakpoint on chromosome 8 was located in an intron of ZFYVE28, which is a negative regulator of epidermal growth factor receptor signalling (Supplementary Figure 2).

For t (6;8), the breakends connected 5′ to 3′ (head of chr6 to tail of chr8) and 3′ to 5′ (tail of chr6 to head of chr8), see Fig. 2b for schematic representation. At the 3′ to 5′ junction, there was a blunt end ligation. The 5′ to 3′ breakpoint junction showed microhomology of 3 bp at 6:27901327–27,901,329 and 8:1266615–1,266,617 (Fig. 2c). For chromosome 8, fifteen bases were lost (8:1266599–1,266,614). A normal copy of chromosome 6 has a length of 170.8 Mb, while a normal copy of chromosome 8 is 139.0 Mb long. The 5′ to 3′ junction resulted in a chromosome length of 140.5 Mb (2.8 Mb from chromosome 6 and 137.7 Mb from chromosome 8), matching the slightly larger derived chromosome 8 in the karyotype picture (Fig. 2a). The 3′ to 5′ junction resulted in a chromosome length of 169.3 Mb (168.0 Mb from chromosome 6 and 1.3 Mb from chromosome 8), matching the slightly shorter derived chromosome 6 in the karyotype picture (Fig. 2a).

We mapped the region of the t (7;14) translocation to 7:118889969/ 14:49733352 and 7:118889973/ 14:49733364. The breakpoint on chromosome 7 was intergenic, while the one on chromosome 14 was located in an intron of the gene CABIN1 (Supplementary Figure 2).

For t (7;14), the breakends connected 5′ to 3′ (head of chr7 to tail of chr14) and 3′ to 5′ (tail of chr7 to head of chr14), for schematic representation see Fig. 3b. At the 3′ to 5′ junction, there was a blunt end ligation. In the 5′ to 3′ junction a micro-insertion of 12 novel bases was observed (Fig. 3c). For chromosome 7, two bases were lost, being 7:118889971–118,889,972. A normal copy of chromosome 7 has a length of 121.8 Mb, while a normal copy of chromosome 14 is 141.8 Mb long. The 5′ to 3′ junction resulted in a chromosome length of 210.9 Mb (118.9 Mb from chromosome 7 and 92.0 Mb from chromosome 14), matching the much larger derived chromosome 7 in the karyotype picture (Fig. 3a). The 3′ to 5′ junction resulted in a chromosome length of 52.7 Mb (3.0 Mb from chromosome 7 and 49.7 Mb from chromosome 14), matching the much shorter derived chromosome 14 in the karyotype picture (Fig. 3a).

### False negative detection of t (1;16)

The RT t (1;16) for pig 7 was not detected in the blind analysis, i.e. false negative (Table 2). After filtering, there was one potential pair of translocations involving chromosome 1 and 16 (1:241933142/ 16:75687308, 1:241932872/ 16:75687319). However, it did not pass the visual inspection, as the breakpoint on chromosome 1 had overlapping forward and reverse reads (like Fig. 5c). RepeatMasker showed that there was a porcine repetitive SINE element of the PRE-1 family [21] on both involved chromosomes at the breakpoint locations (1:241932894–241,933,143 and 16:75687060–75,687,314), causing the discordant pairs and split alignments. In addition, these inter-chromosomal translocations showed up in the sequences of several other pigs indicating it is a common rearrangement due to the repetitive element and not the RT.

After unblinding, we investigated all translocations involving chromosome 1 and 16 from the raw DELLY output, because the RT may have been reported by DELLY, but may not have fulfilled all filtering criteria. DELLY detected 375 inter-chromosomal translocations between chromosome 1 and 16. Among those, we selected the 56 translocations with a matching reciprocal translocation with matching connection type (3′ to 3′ and 5′ to 5′ or 3′ to 5′ and 5′ to 3′), resulting in 28 possible RT pairs. For only two of those pairs, both breakends on the same chromosome were within 100 bp from each other. One had overlapping forward and reversed reads (like Fig. 5c) on chromosome 1, and was present in multiple animals, hence unlikely to be the true RT. While the other showed good characteristics of an RT on chromosome 1 (197320432–97,320,434), and a potential RT breakpoint in a repetitive region on chromosome 16 (16:23532186–23,532,204; Fig. 4b). Hence we investigated this pair further.

Based on the DELLY output, one translocation (1:97320434/ 16:23532200) was mapped with high quality and passed de criteria, but the other translocation (1:97320432/ 16:23532204) had a low mapping quality of 12, only 3 discordant read pairs and no split alignments to support the junction. Looking at it visually (Fig. 4b), there was actually sufficient support for an inter-chromosomal translocation at 1:97320431 and 16:23522204, supported by split alignments partially mapping to each location. However, most forward reads were discordantly paired with a mate mapping to other locations on the genome due to a Pre0_SS element on chromosome 16 at 23532226–23532486. The L1MD element (at 16:23532134–23,532,196) did not seem to hamper the detection of the RT breakpoint. The Pre0_SS element led to the low quality and imprecise labels in the DELLY output. This background due to the repetitive element of Pre0_SS was also observed in other pigs at this location on chromosome 16, but without the split alignments supporting the RT for Pig7. This indicates that the blind analysis of DELLY output in combination with the filtering criteria were not suitable to detect this RT located in a repetitive region.

We mapped the region of the t(1;16) translocation to 1:97320431/ 16:23532204 and 1:97320436/ 16:23532186. The breakpoint on chromosome 1 was intergenic, while the one on chromosome 16 was located in an intron of the gene EGFLAM (Fig. 4b).

For t (1;16), the breaks connected 5′ to 3′ (head of chr1 to tail of chr16) and 3′ to 5′ (tail of chr1 to head of chr16), for schematic representation see Fig. 4c. At both junctions, there were micro-insertions of 15 and 20 novel bases (Fig. 4d). For chromosome 1, four bases were lost being 1:97320432–97,320,435. For chromosome 16 seventeen bases were lost, being 16:23532187–23,532,203. A normal copy of chromosome 1 has a length of 274.3 Mb while a normal copy of chromosome 16 is 79.9 Mb long. The 5′ to 3′ junction resulted in a chromosome length of 153.7 Mb (97.3 Mb from chromosome 1 and 56.4 Mb from chromosome 16), matching the larger derived chromosome 16 in the karyotype picture (Fig. 4a). The 3′ to 5′ junction resulted in a chromosome length of 200.5 Mb (23.5 Mb from chromosome 1 and 177.0 Mb from chromosome 16), matching the shorter derived chromosome 1 in the karyotype picture (Fig. 4a).

### Reducing sequencing coverage

To test the impact of sequencing coverage on the ability to discover the RT, we randomly reduced the sequencing coverage of the carriers to 10, 15, and 20 fold coverage. This had a big impact on the blind detection of the RTs (Supplementary Table 1). With 10 fold coverage not all the actual RT breakpoints were detected by DELLY (pig 1, 2, 6), and many of the ones that were detected had a low quality label (pig 3, 4, 5). Only the RT of pig 5 could be detected with relaxed filtering criteria. This indicates that 10 fold coverage is not enough for proper detection of RT.

With 15 fold coverage only the RT for pig 5 was detected, however for 4 other carriers both breakpoints were initially detected by DELLY but lost due to filtering. The filtering criteria for samples with 30 fold coverage can be set stringent to remove as many non RT events as possible, for lower fold coverage these filtering criteria might be too stringent. Therefore we relaxed the filtering criteria, mainly with respect to quality and number of split reads, and then the RT could be detected with 15 fold coverage for 5 out of the 7 carriers (Supplementary Table 1).

With 20 fold coverage all the RTs were detected by DELLY, but still many were of low quality and hence filtered out using the strict filtering criteria (Supplementary Table 1). Relaxing the filtering criteria resulted in the detection of 6 of the 7 RT carriers (Supplementary Table 1), which is just as good as with 30 fold coverage (Table 2). These results show that 20 fold coverage is a minimum coverage to detect RTs, and that filtering criteria should be adjusted to sequencing coverage.

## Discussion

The aim of this study was to test the possibilities of using paired end short read sequencing for (blind) detection of balanced RTs and investigate the breakpoints and junctions of the translocations. The results showed that it is possible to detect balanced RTs using short reads. We recovered 6 out of 7 carriers and all 15 non-carriers came out negative. The results are similar to studies in human and show the potential of sequence data for detection of RTs.

In our study the exact RT breakpoints were not known from the karyotyping. We only knew the two non-homologous chromosomes involved in the RT and had a picture of the chromosome staining indicating the difference in size of the derived and normal chromosomes. We considered it unlikely to falsely end up with two matching inter-chromosomal translocations that meet all filtering criteria and comprise exactly the two non-homologous chromosomes involved in the RT. In addition, we reconstructed the junctions based on split alignments and compared the size of the resulting derived chromosomes to the normal chromosomes, which roughly matched with the Giemsa stained pictures of the karyotype for the three unique RTs detected in the sequence data of the carriers. Furthermore, the related individuals with t (2;4) provided the exact same final RT position suggesting that the method is reproducible when samples are sequenced again. However, reducing the coverage showed that 20 fold coverage is minimally needed.

One RT was not detected using the blind screening (Pig 7 with t (1;16)), however, a highly likely RT was discovered investigating all inter-chromosomal translocations between the two non-homologous chromosomes involved after unblinding. This RT was located in a repetitive region, showing the limitations of short read sequence data, as short reads are not able to span repeats. On the other hand, long read sequencing is emerging as a strong technique for detection of RT in complex regions [12, 13]. Although still expensive, long read sequencing is suited for any type of structural variance detection [11], and might become the standard in the future. Long read sequencing is also currently actively used to improve the reference genomes. The accuracy of the reference genome is another important factor for the success of routine RT screening. Although the *Sus scrofa* genome build 11.1 is highly complete [22], we ignored the 583 unmapped contigs, which could be involved in RTs. A complete and accurate (pan) reference genome will benefit structural variation detection in general.

With the development of accurate (pan) reference genomes and reducing costs of sequencing, we can use sequence data at sufficient coverage to detect all kinds of structural variation normally detected by karyotyping, including Robertsonian translocations, aneuploidy, inversions, copy number variants, and even mosaicism of male and female cells, suggesting sequence data can replace karyotype screening. It is certainly easier to obtain DNA material for sequencing than to perform chromosome staining with laborious analysis of lymphocyte cultures. Furthermore, sequence data provides an enormous amount of additional genomic information besides large karyotype defects, because it is possible to detect recessive lethal alleles [23], de novo events and smaller structural variation events [5], as well as SNP and InDels.

Bioinformatic tools currently available can detect a large range of structural variation in sequence data, and many are able to detect inter-chromosomal translocations [24]. For our specific goal we preferred a tool that detects the precise location of the breakpoints at a base pair resolution, and therefore uses split reads, and had a high specificity to assure the RT breakpoints are detected. Among the available structural variation callers, there are a number of so called hybrid callers that combine different types of anomalously mapped reads to increase the calling sensitivity, e.g. DELLY, Meerkat, and SoftSV [24]. Each have their own features to perform best for certain structural variants under certain sequencing conditions [24].

The short read sequence data we used here had sufficient split alignments for detailed analysis of the breakpoint junctions because almost the whole length of the DNA fragments were sequenced with 30 fold coverage. The average median insert size was 307 bp, although rather short for detection of breakpoints in repetitive elements, it was beneficial for detailed analysis of detected breakpoint junctions. With paired end reads of 150 bp in size, the inner distance of the fragments was very small which leads to more reads crossing the breakpoint than spanning the breakpoint [24]. The detailed analysis of the breakpoints and junctions suggested three junctions showing microhomology, three junctions with blunt-end ligation, and three micro-insertions at the breakpoint junctions. This is in line with the type of breakpoint junctions known from a large scale human study: 45% blunt-end ligations, 29% microhomology, 25% micro-insertions, and only 1% long stretches of homologous sequences [15].

We also observed disruption of the genes *ENSSSCG00000032003* (orthologous to human *BRCA2*), *C1orf112*, *SLC9A5*, *ZFYVE28*, *CABIN1*, and *EGFLAM*. The *BRCA2* ortholog is known to cause several forms of cancer according to the OMIM entry 600,185 [20]. Homozygous knockout for *BRCA2* in mice caused embryonic lethality, however in heterozygous state, like the RT carriers, they did not reveal strong phenotypic associations [25]. The gene *EGFLAM* has been associated with Adiaspiromycosis and Muscular Dystrophy-Dystroglycanopathy. Homozygous knockout mice were vital and showed similar phenotypes as wild types and heterozygous individuals, except for impaired vision [26]. The gene *CABIN1* is located ~400Kb from *BCR*, breakpoint cluster region, which is involved in the Philadelphia translocation t (9;22) resulting in a fusion gene in leukaemia cancer cells in humans. These gene disruptions did not lead to phenotypic abnormality, perhaps because the gene disruption is present in heterozygous state in RT carriers, or because phenotypic onset did not occur at this early age (tested individuals are less than 2 year old). Gene disruptions are also common in human RTs [15], with a high frequency of disruptions of disease related genes in patients [27].

In this study, all RTs seemed to be inherited rather than de novo. The expectation is that RT carriers have reduced number of (liveborn) piglets because half of their gametes are unbalanced, leading to unbalanced foetuses, which are most likely unviable. For pig 2, 5, and 7 this was indeed the case for one of the parents, or the individual itself (pig 7). Surprisingly, for the related t (2, 4) carriers pig 1, 3, 4, and 6, there was no clear reduction in litter size from their parents. Given their pedigree, the dam of pig 3, 4, and 6, and dam of pig 1 (maternal granddam of pig 3, 4, and 6) are most likely carriers of t (2,4) (Supplementary Figure 1). However, these dams and none of the sires involved showed a reduced litter size. This might explain why this RT still segregates in the population. The average liveborn litter size of the dams were based on only 5 to 6 litters, and could be average by chance. The number of live born includes piglets that survived the first 24 h after birth. It may be that the unbalanced piglets of the t (2, 4) carriers survived the first 24 h. However, we observed only 3 and 4 piglets across the litters of the those dams, respectively, that deceased within the first week. We checked genotype array data of offspring of these dams if there were any unbalanced viable offspring, but this was not the case. If viability of unbalanced zygotes is affected very early in zygote development, other embryos might take over the open space in the placenta because on average around 20–30 ovulations take place per cycle [28] and very likely all of them are fertilized. However, implantation is afterwards the main bottle neck and enough viable embryos will still be available to occupy free spaces in the uterus. Although a 50% reduction in litter size is expected, literature suggests highly variable ranges of reduction in average litter size of RT carriers, i.e. between 10 and 100%, with an average of ~ 40% [1, 3, 4, 7]. Hence, it is observed more often that RTs show limited reduction in litter size.

## Conclusion

In this study we showed that paired end short read sequencing can be used for blind detection of balanced reciprocal translocations in pigs if sequencing depth is at least 20 fold coverage. The sequence data provided insight in the precise location and features of the breakpoint junctions of the translocations. However, translocations in repetitive areas may require larger fragments or even long read sequence data.

## Methods

### Reciprocal translocation carriers

Reciprocal translocation carriers were identified by routine screening for abnormal karyotypes that is in place at Topigs Norsvin (Beuningen, the Netherlands) for boars used for AI. The screening is done with Giemsa banding technique to produce a visible karyotype by Giemsa staining of condensed metaphase chromosomes (performed by VHLGenetics, Wageningen, the Netherlands). Detected carriers for which DNA material was available were selected, which resulted in 7 male carriers with RTs. The Giemsa stained pictures of the karyotype of the carriers are in Figs. 1a, 2a, 3a and 4a, with the RT position indicated.

After DNA extraction from the samples, the DNA was sequenced with Illumina HiSeq 150PE sequencing at 30 fold coverage. The sequence reads were trimmed using sickle [29] and aligned against *Sus scrofa* genome build 11.1 (GCA_000003025.6) using BWA mem [30]. The bam files were sorted, indexed and duplicate marked using samtools [31]. Finally GATK was used for re-alignment using RealignmentTargetCreator and IndelRealigner [32].

The average sequencing coverage of the carriers was 33.9 fold (30.3–37.9 fold). On average, 99.0% (97.6–99.3%) of the reads could be mapped to the reference genome. The median insert size of the reads was on average 307 bp (303-311 bp).

The animals were not euthanised for this study. Blood was drown for karyotyping and DNA was extracted from either blood, semen or a tissue sample. All sampling was routine procedures in the breeding program.

### Non-carriers

An independent set of 15 non-carrier control pigs were sequenced at 30 fold. These were all normal males that underwent the same routine karyotype screening with chromosome staining as the carriers.

Sequence alignment was exactly the same as described above for the carriers. The average sequencing coverage of the non-carriers was 30.3 fold (28.8–34.3 fold). On average, 98.6% (97.5–99.3%) of the reads could be mapped to the reference genome. The median insert size of the reads was on average 304 bp (246-334 bp).

### Calling inter-chromosomal translocations

DELLY v2 was used to identify inter-chromosomal translocations. DELLY is an integrated structural variant detection program, using paired ends, split reads and read depth to discover genomic rearrangements in the genome [33]. DELLY with default settings was used for germline structural variation calling by sample. Unplaced contigs and mitochondrial DNA were excluded from the analysis. Although DELLY can detect a variety of structural variants, we only analysed the samples for inter-chromosomal translocations (Breakend (BND)).

With 30 fold coverage we assumed to find translocations that passed the quality filter set by DELLY (PASS: PE/SR support = 3 or more and mapping quality> 20), and that they have been refined using split reads indicated by the PRECISE tag in DELLY output (referred to as basic filtering in tables). Next filtering (referred to as final filtering in tables) was based on maximum mapping quality (MAPQ = 60), at least 10 split reads supporting the translocation, not more than 60 discordant paired end reads (2 times intended coverage), consensus alignment quality (SRQ) bigger than 0.9 (where 1 indicates 100% identity to the reference), and matching second (reciprocal) translocation with matching connection type (3′ to 3′ and 5′ to 5′ or 3′ to 5′ and 5′ to 3′), because we were after RTs only.

### Visual classification of breakpoints

After filtering, the remaining translocations required further classification into none RT events and possible RT translocation by visualising the breakpoints in IGV [19]. A RepeatMasker track was added to visualise the positions of known repetitive elements. During this manual inspection we discovered that the remaining possible RTs show 3 different patterns on the chromosomes involved. Figure 5 shows the three types observed, with possible RT translocations (Fig. 5a) showing good reads of the intact chromosome as well as discordant read pairs and split reads at the two breakends from the affected chromosome. The other two observed patterns showed patterns typical for repetitive elements (Fig. 5b and c), which was confirmed by RepeatMasker. A true RT showed two patterns like Fig. 5a, one on each chromosome involved. This clear distinction made it possible to identify true RTs and none RT events. The non RT events are likely to be correctly detected as inter-chromosomal translocations by DELLY, but are mostly inter-chromosomal breakpoints due to repetitive elements segregating on multiple chromosomes and not due to an RT event.

### Analysis of breakpoints and junctions

Chromosome junctions were analysed using the sequence reads at the breakpoints of the detected RTs. The sequence reads with split alignment contained the sequence at junctions. Based on the forward and reverse sequence reads spanning the two junctions, we could reconstruct the junctions. By doing so, we could confirm the connection type identified by DELLY and investigated whether bases were lost or gained at the junctions. In addition, we checked if there were any genes involved in the translocations.

### Reducing sequencing coverage

To test the impact of sequencing coverage on the ability to discover the RT, we reduced the sequencing depth of the carriers to 10, 15, and 20 fold coverage and reran the pipeline to detect the RTs. For each case, the sequencing depth was reduced by random selection of 30, 50, and 60% of the trimmed reads. These were aligned to the reference genome following the same steps as described above and screened for RTs using DELLY as described above.

Since the filtering criteria were chosen based on 30 fold coverage, we also applied a less stringent filtering more suitable for lower coverage samples to filter non RT events. Translocations that failed the quality filter of DELLY (LowQual instead of PASS) and with 5 or more split reads were retained.

## Supplementary information

**Additional file 1: Figure S1.** Pedigree of t (2, 4) carriers Pig 1, 3, 4, and 6. Circles represent females, squares represent males. The red square indicates positive t (2, 4) reciprocal translocation carrier based on Giemsa staining, whereas, pink circles indicate likely t (2, 4) carrier parent that transmitted the RT. **Table S1.** Results of reduced sequencing depth, with both strict and relaxed filtering criteria. **Figure S2.** IGV images of the aligned reads at the breakends on both chromosomes involved in the reciprocal translocation of each detected RT in 6 of the 7 carriers. Grey reads are normal reads. Colored reads are reads of discordant pairs (mate maps to another chromosome). Green (A), red (T), blue (C) and orange/brown (G) bases show mismatched bases from split reads. In addition, an Ensemble gene spans track (gene positions indicated with blue bars) and RepeatMasker track (repetitive elements indicated with green bars) are given at the top.

## Data Availability

Data are available from the authors upon reasonable request and with permission of Topigs Norsvin, requests can be directed to aniek.bouwman@wur.nl. The *Sus scrofa* 11.1 reference genome used for this study has GenBank assembly accession number GCA_000003025.6.

## Abbreviations

AI: Artificial insemination
DNA: Deoxyribonucleic acid
RT: Reciprocal translocation
SINE: Short interspersed nuclear element
SNP: Single nucleotide polymorphism
