# Supplementary material for: Using short read sequencing to characterise balanced reciprocal translocations in pigs
Source: BMC Genomics. 2020 Aug 24;21:576. doi: 10.1186/s12864-020-06989-x (PMC7444045; doi:10.1186/s12864-020-06989-x)
Supplement: Supplementary file 1 — Additional file 1: Figure S1. Pedigree of t (2, 4) carriers Pig 1, 3, 4, and 6. Circles represent females, squares represent males. The red square indicates positive t (2, 4) reciprocal translocation carrier based on Giemsa staining, whereas, pink circles indicate likely t (2, 4) carrier parent that transmitted the RT. Table S1. Results of reduced sequencing depth, with both strict and relaxed filtering criteria. Figure S2. IGV images of the aligned reads at the breakends on both chromosomes involved in the reciprocal translocation of each detected RT in 6 of the 7 carriers. Grey reads are normal reads. Colored reads are reads of discordant pairs (mate maps to another chromosome). Green (A), red (T), blue (C) and orange/brown (G) bases show mismatched bases from split reads. In addition, an Ensemble gene spans track (gene positions indicated with blue bars) and RepeatMasker track (repetitive elements indicated with green bars) are given at the top. [file 12864_2020_6989_MOESM1_ESM.docx]

# Supplementary material

Using short read sequencing to characterise balanced reciprocal translocations in pigs - Bouwman et al.

Supplementary Figure 1 – page 1

Supplementary Table 1 – page 2

Supplementary Figure 2 – page 3-5


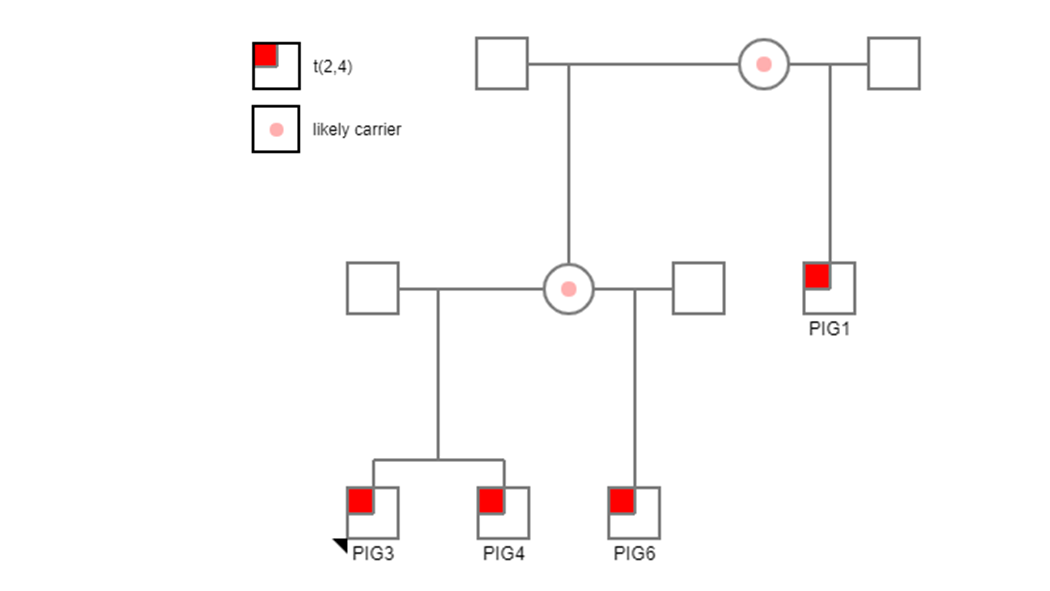


**Supplementary Figure 1.** Pedigree of t(2,4) carriers Pig 1, 3, 4, and 6. Circles represent females, squares represent males. The red square indicates positive t(2,4) reciprocal translocation carrier based on Giemsa staining, whereas, pink circles indicate likely t(2,4) carrier parent that transmitted the RT.

**Supplementary Table 1**. Results of reduced sequencing depth, with both strict and relaxed filtering criteria

| Animal |  | PIG1 | PIG2 | PIG3 | PIG4 | PIG5 | PIG6 | PIG7 |
| --- | --- | --- | --- | --- | --- | --- | --- | --- |
| Karyotype | | t(2;4) | t(6;8) | t(2;4) | t(2;4) | t(7;14) | t(2;4) | t(1;16) |
| 10 fold | Coverage | 10.2 | 11.9 | 10.5 | 11.0 | 11.7 | 9.8 | 9.5 |
| Strict^1^ | DELLY out | 18,772 | 26,448 | 22,308 | 25,634 | 18,809 | 14,042 | 20,364 |
| filtering | Basic filt | 146 | 267 | 166 | 168 | 209 | 139 | 146 |
|  | Final filt^3^ | 4(2) | 6(3) | 0 | 2(1) | 4(2) | 0 | 4(2) |
|  | Visual insp | 0 | 0 | 0 | 0 | 0 | 0 | 0 |
|  |  |  |  |  |  |  |  |  |
| 10 fold | Basic filt | 2739 | 3939 | 3007 | 3084 | 3675 | 2710 | 2831 |
| Relaxed^2^ | Final filt^3^ | 32(16) | 24(12) | 28(14) | 26(13) | 40(20) | 8(4) | 28(14) |
| filtering | Visual insp | 0 | 0 | 0 | 0 | 1(7,14) | 0 | 0 |
|  |  |  |  |  |  |  |  |  |
| 15 fold | Coverage | 16.8 | 19.5 | 17.2 | 18.1 | 19.2 | 16.1 | 15.6 |
| Strict^1^ | DELLY out | 34556 | 46817 | 40754 | 45934 | 44923 | 35662 | 37186 |
| filtering | Basic filt | 405 | 598 | 463 | 479 | 548 | 423 | 429 |
|  | Final filt^3^ | 8(4) | 16(8) | 12(6) | 18(9) | 24(12) | 4(2) | 14(7) |
|  | Visual insp | 0 | 0 | 0 | 0 | 1(7,14) | 0 | 0 |
|  |  |  |  |  |  |  |  |  |
| 15 fold | Basic filt | 5854 | 7769 | 6316 | 6537 | 7465 | 5738 | 6009 |
| Relaxed^2^ | Final filt^3^ | 46(23) | 62(31) | 46(23) | 54(27) | 60(30) | 44(22) | 52(26) |
| filtering | Visual insp | 0 | 1(6,8) | 1(2,4) | 1(2,4) | 1(7,14) | 1(2,4) | 0 |
|  |  |  |  |  |  |  |  |  |
| 20 fold | Coverage | 20 | 23.3 | 20.5 | 21.6 | 22.9 | 19.2 | 18.6 |
| Strict^1^ | DELLY out | 33034 | 46672 | 39783 | 45198 | 44767 | 34398 | 35871 |
| filtering | Basic filt | 569 | 697 | 549 | 621 | 717 | 554 | 564 |
|  | Final filt^3^ | 20(10) | 28(14) | 16(8) | 26(13) | 30(15) | 6(3) | 30(15) |
|  | Visual insp | 0 | 0 | 0 | 0 | 1(7,14) | 0 | 0 |
|  |  |  |  |  |  |  |  |  |
| 20 fold | Basic filt | 7,323 | 9,586 | 7,869 | 8,215 | 9,505 | 7,210 | 7,477 |
| Relaxed^2^ | Final filt^3^ | 54(27) | 68(34) | 48(24) | 66(33) | 62(31) | 50(25) | 54(27) |
| filtering | Visual insp | 1(2,4) | 1(6,8) | 1(2,4) | 1(2,4) | 1(7,14) | 1(2,4) | 0 |

^1^ Strict filtering: Basic filtering: Pass DELLY quality filter and precise position; final filtering: mapping quality=60, at least 10 split reads supporting the translocation, ≤60 discordant paired end reads, consensus alignment quality>0.9, and matching second translocation with matching connection type present

^2^ Relaxed filtering: **Translocations that failed the quality filter of DELLY (LowQual instead of PASS; basic filtering) and with 5 or more split reads** supporting the translocation (final filtering) **were retained.**

^3^ one of the filter criteria for the detected inter-chromosomal translocations was the presence of a matching pair, hence between brackets are the number of reciprocal pairs, i.e. the actual number of possible RTs

| Pig1  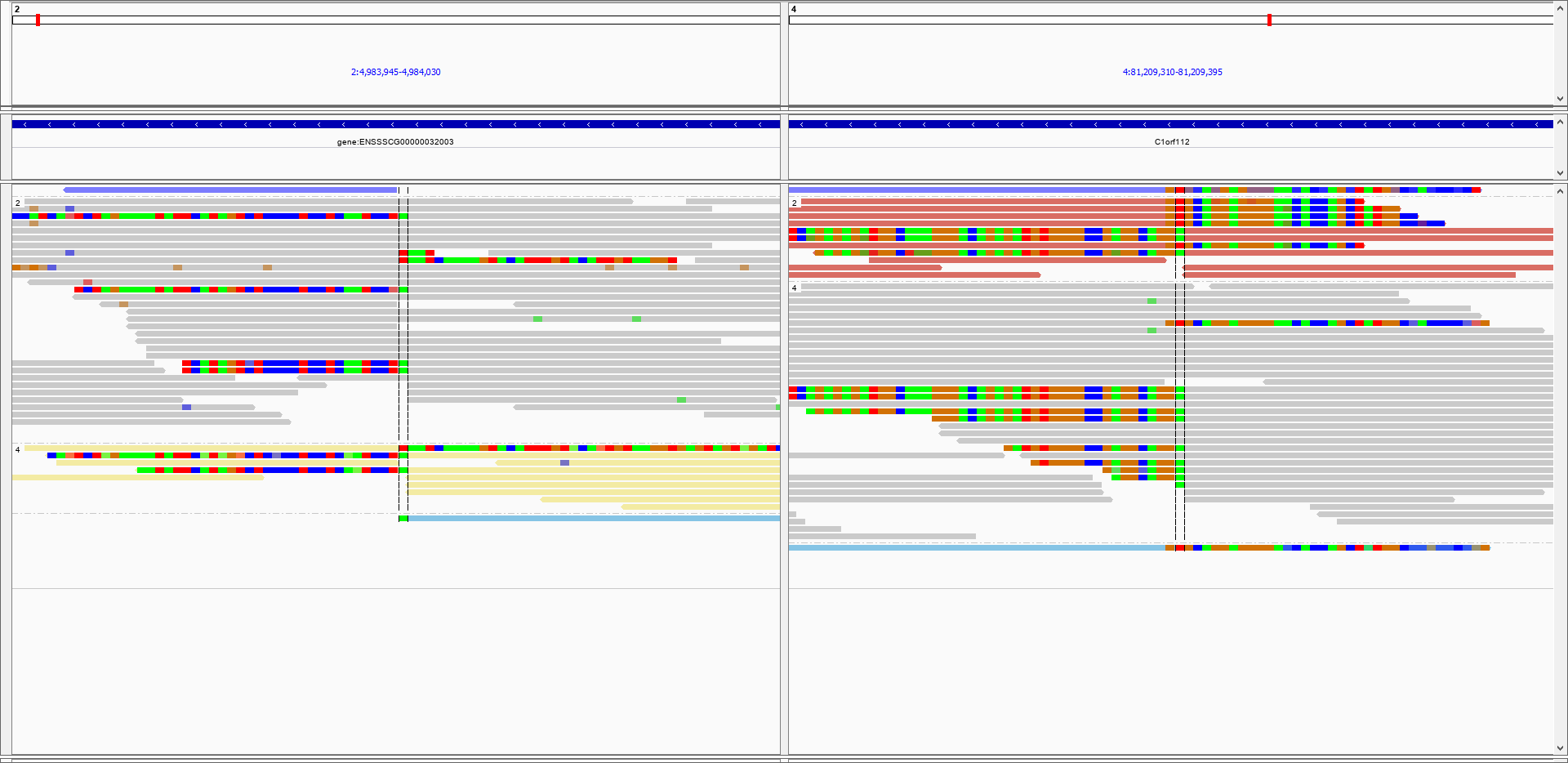 |
| --- |
| Pig2  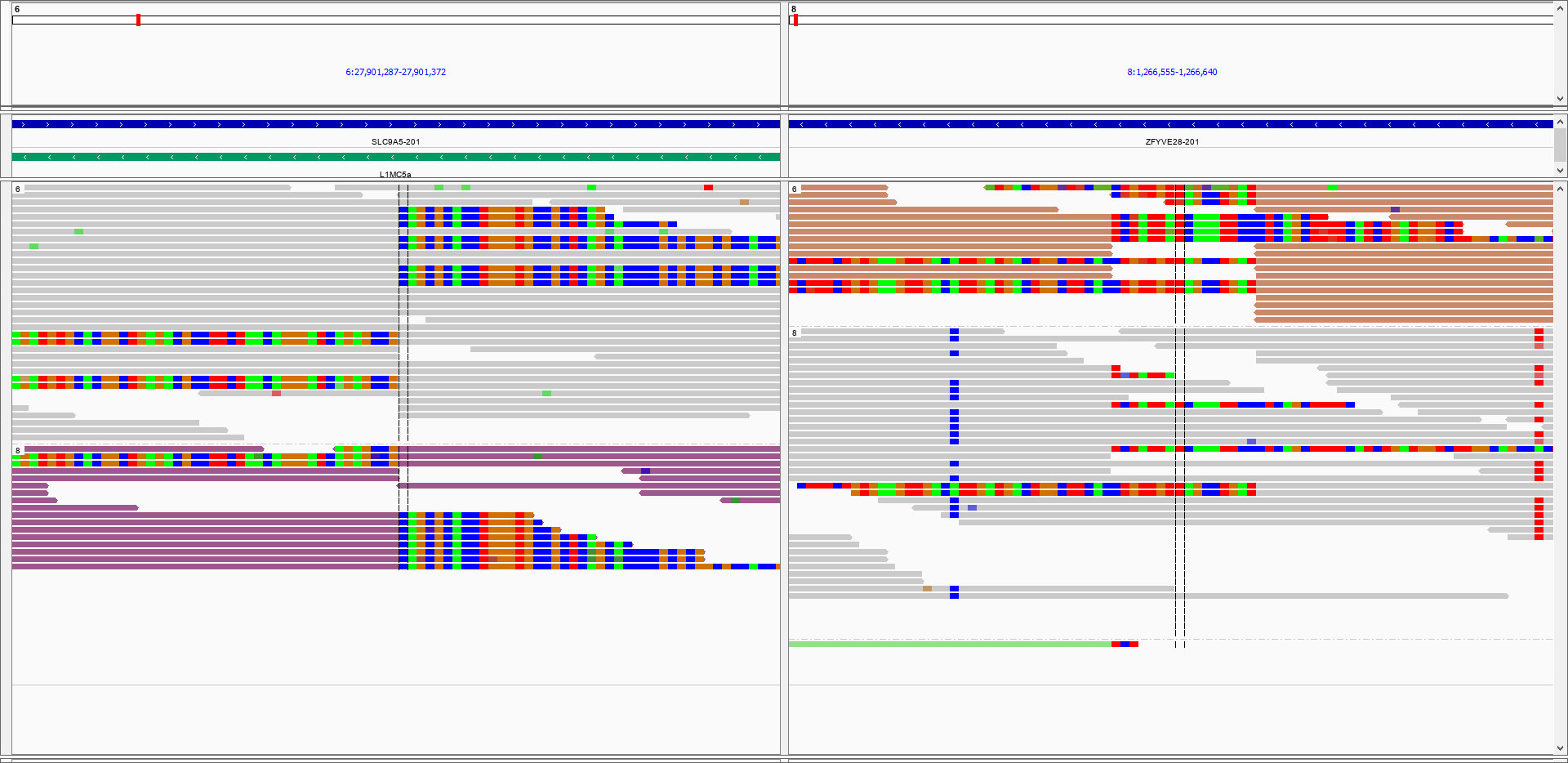 |
| Pig3  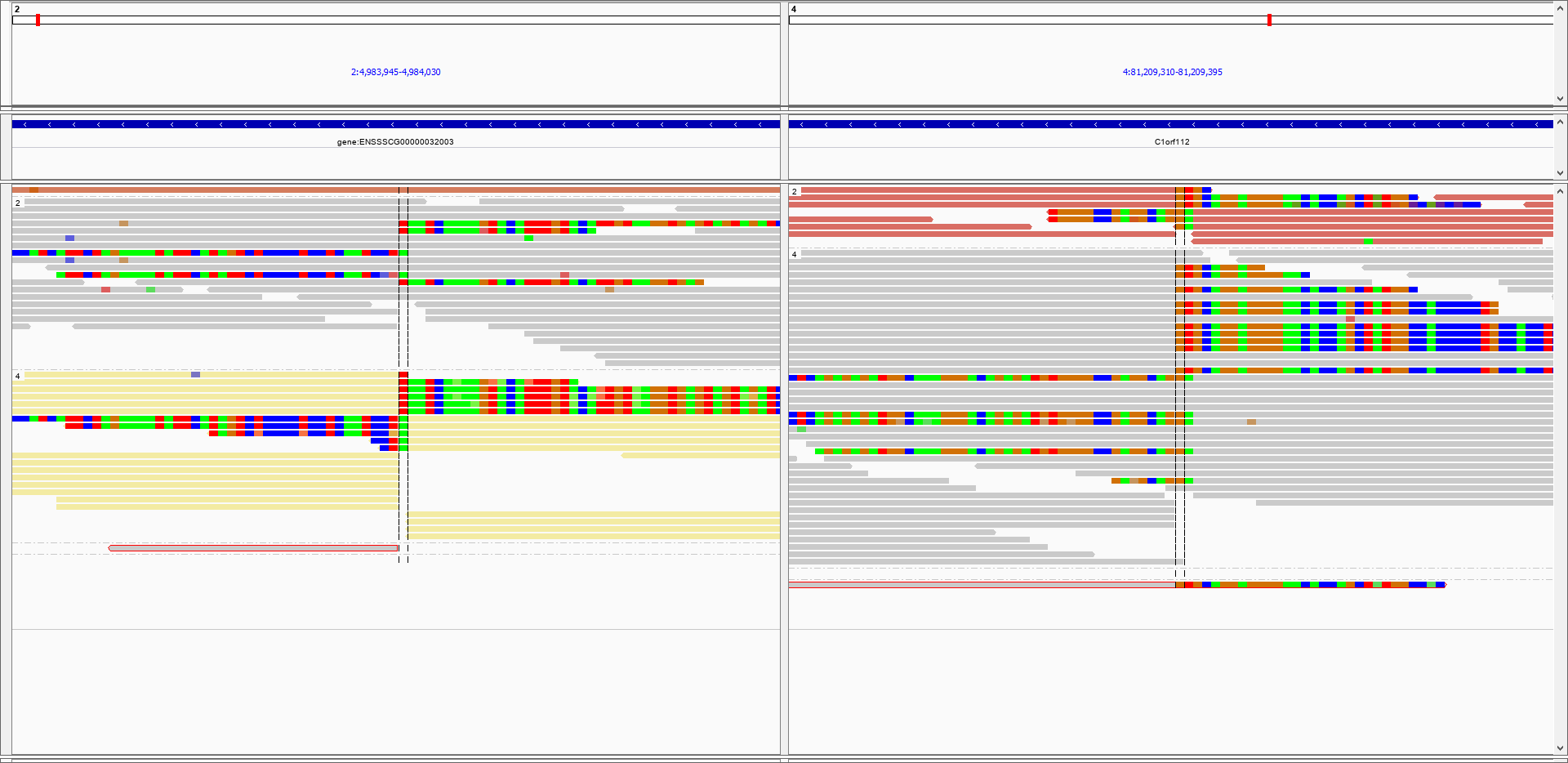 |

| Pig4  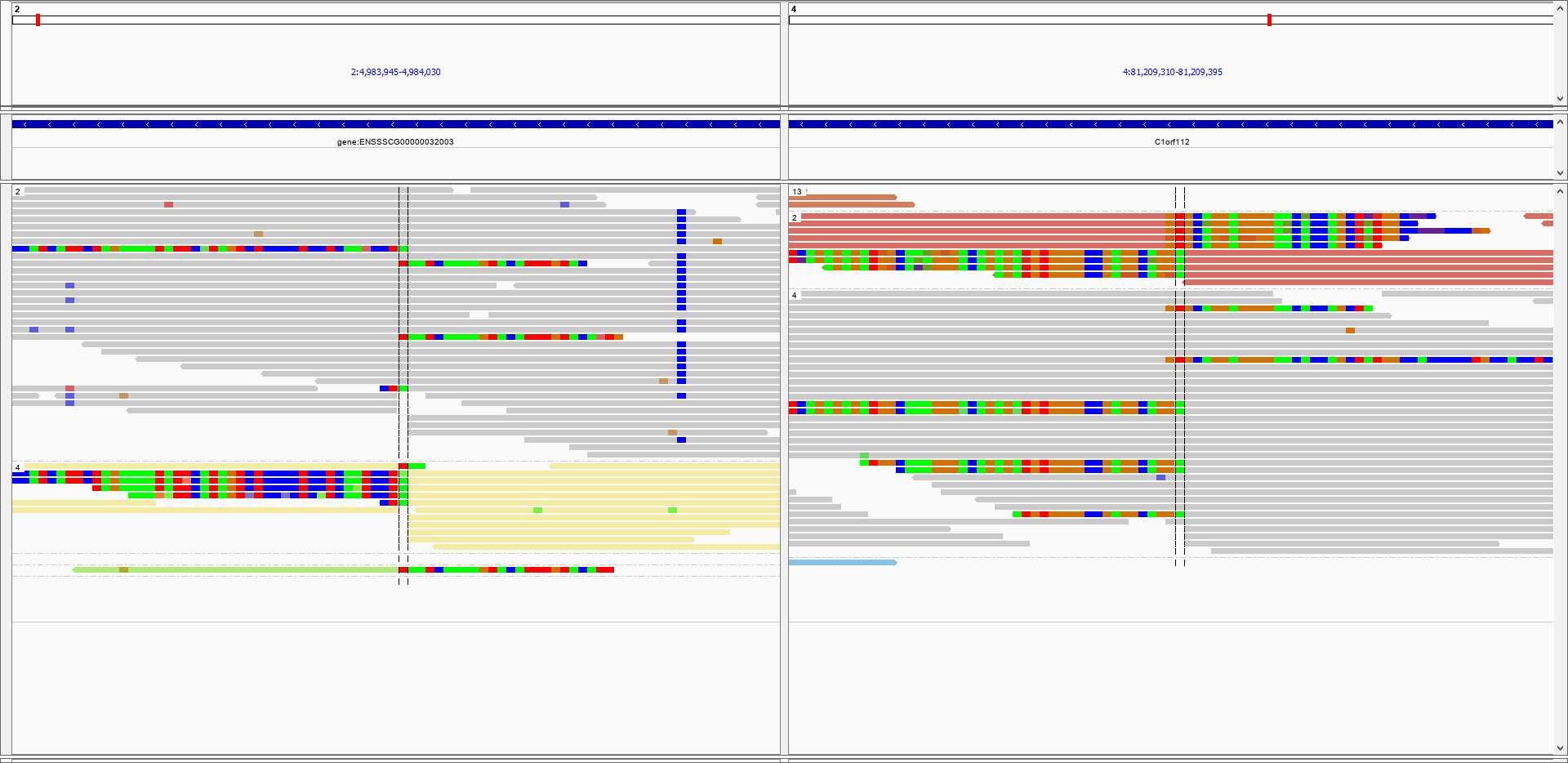 |
| --- |
| Pig5  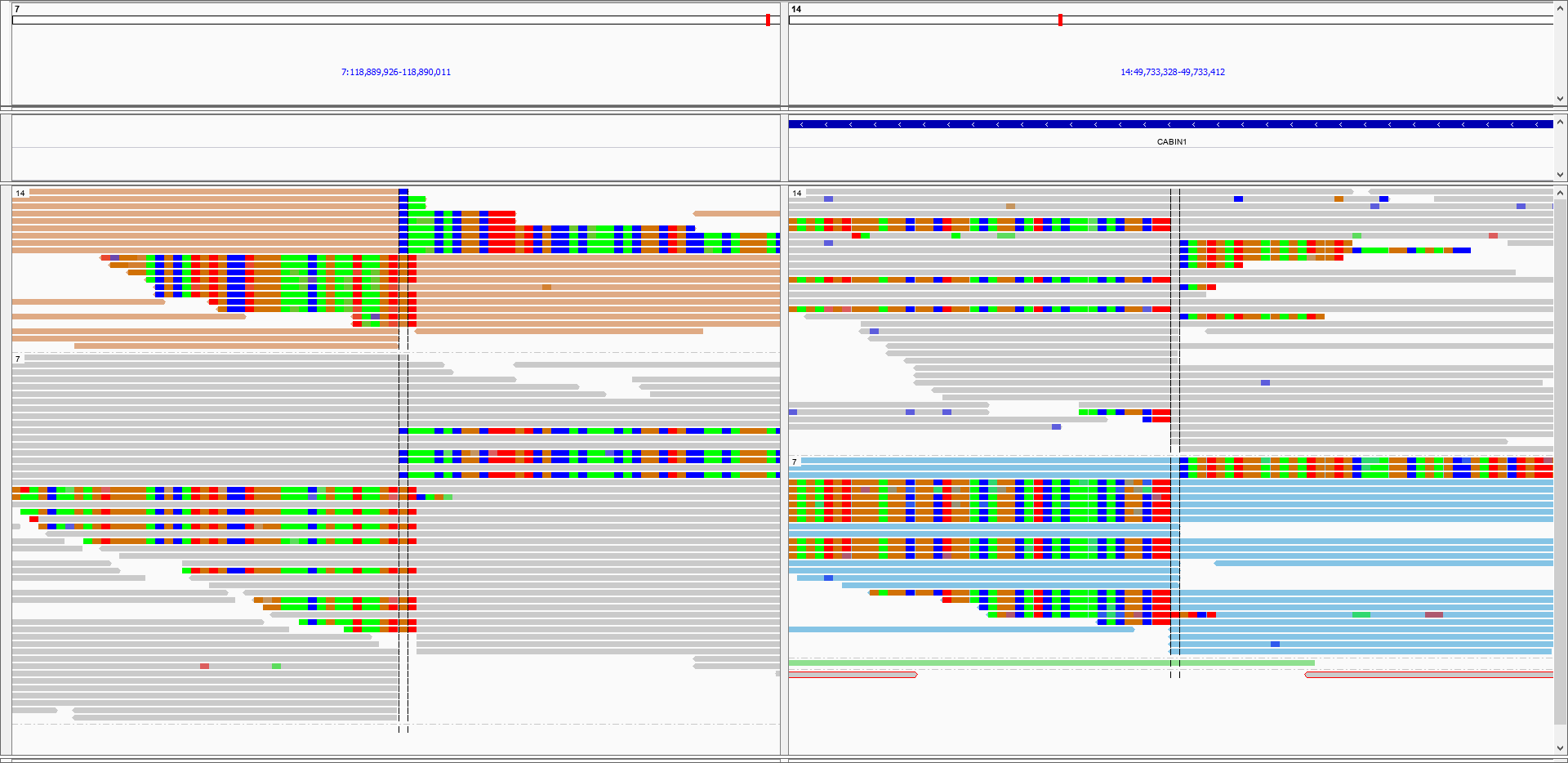 |
| Pig6  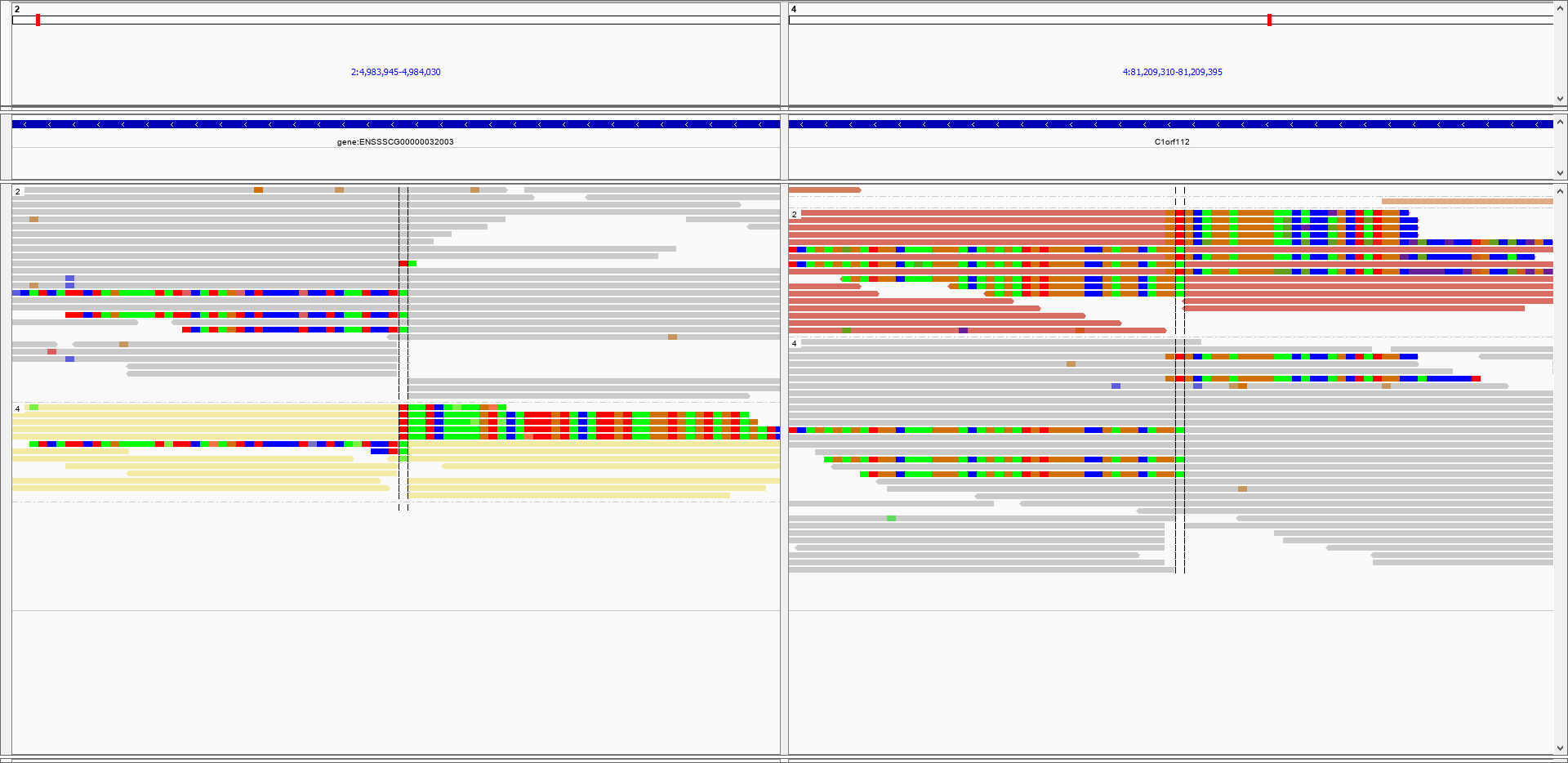 |

**Supplementary Figure 2.** IGV images of the aligned reads at the breakends on both chromosomes involved in the reciprocal translocation of each detected RT in 6 of the 7 carriers. Grey reads are normal reads. Colored reads are reads of discordant pairs (mate maps to another chromosome). Green (A), red (T), blue (C) and orange/brown (G) bases show mismatched bases from split reads. In addition, an Ensemble gene spans track (gene positions indicated with blue bars) and RepeatMasker track (repetitive elements indicated with green bars) are given at the top.
